# Deletion of the HAMP domains from the histidine kinase CaNik1p of *Candida albicans* or treatment with fungicides activates the MAP kinase Hog1p in *S. cerevisiae* transformants

**DOI:** 10.1186/1471-2180-13-209

**Published:** 2013-09-17

**Authors:** Mohammed El-Mowafy, Mahmoud M Bahgat, Ursula Bilitewski

**Affiliations:** 1 AG Biological Systems Analysis, Helmholtz Centre for Infection Research (HZI), Inhoffenstr. 7, 38124 Braunschweig, Germany; 2 Present address: Department of Microbiology and Immunology, Faculty of Pharmacy, Mansoura University, Mansoura, Egypt; 3 Therapeutic Chemistry Department, Immunology and Infectious Diseases Research Group, the National Research Center, Cairo, Egypt; 4 Present address: Research Group Biomarkers for Infectious Diseases, Institute for Experimental Infection Research, TWINCORE, Centre for Experimental and Clinical Infection Research, Feodor-Lynen-Str. 7, 30625 Hannover, Germany

**Keywords:** Group III histidine kinase, CaNik1p, Point mutations, Protein domains, Fludioxonil, Transformed *Saccharomyces cerevisiae*, HAMP domains

## Abstract

**Background:**

Microorganisms use two-component signal transduction (TCST) systems to regulate the response of the organism to changes of environmental conditions. Such systems are absent from mammalian cells and are thus of interest as drug targets. Fungal TCST systems are usually composed of a hybrid histidine kinase, comprising the histidine kinase (HisKA) domain and a receiver domain, a histidine phosphotransfer protein and a response regulator. Among the 11 groups of fungal histidine kinases, group III histidine kinases are of particular relevance as they are essential for the activity of different groups of fungicides. A characteristic feature is the N-terminal amino acid repeat domain comprising multiple HAMP domains, of which the function is still largely unknown. In *Candida albicans,* a fungal human pathogen, three histidine kinases were identified, of which CaNik1p is a group III histidine kinase. Heterologous expression of this protein in *Sacchromyces cerevisiae* conferred susceptibility to different fungicides. Fungicide activity was associated with phosphorylation of the mitogen activated protein kinase Hog1p.

**Results:**

We have constructed mutated versions of CaNik1p, from which either all HAMP domains were deleted (CaNik1pΔHAMP) or in which the histidine kinase or the receiver domains were not-functional. Expression of *CaNIK1*Δ*HAMP* in *S. cerevisiae* led to severe growth inhibition. Normal growth could be restored by either replacing the phosphate-accepting histidine residue in CaNik1pΔHAMP or by expressing *CaNIK1*Δ*HAMP* in *S. cerevisiae* mutants, in which single genes encoding several components of the HOG pathway were deleted. Expression of proteins with non-functional histidine kinase or receiver domains resulted in complete loss of susceptibility to antifungals, such as fludioxonil. Conditions leading to growth inhibition of transformants also led to phosphorylation of the MAP kinase Hog1p.

**Conclusion:**

Our results show that functional histidine kinase and receiver domains of CaNik1p were essential for antifungal susceptibility and for activation of the Hog1p. Moreover, for the first time we show that deletion of all HAMP domains from CaNik1p led to activation of Hog1p without an external stimulus. This phenotype was similar to the effects obtained upon treatment with fungicides, as in both cases growth inhibition correlated with Hog1p activation and was dependent on the functionality of the conserved phosphate-accepting histidine residue.

## Background

Immune-compromised patients are at high risk of becoming infected by opportunistic fungi, such as *Candida* and *Aspergillus sp*. *Candida sp*. are the fourth most frequent cause of hospital acquired blood stream infections and up to 90% of HIV patients receive mucosal candidiasis at least once [1]. Although infections with non-*albicansCandida sp*. have emerged in recent years [2], the species *C. albicans* is still responsible for the majority of the cases [3,4].

Several antifungals are available in the market, yet, toxicity and/or development of resistance represent major concerns [5]. Among these is the former “gold standard” therapeutic amphotericin B that invariably causes toxicity in patients, negating the importance of its fungicidal activity. Although azoles and echinocandins represent the most widely used treatments of candidiasis, the acquisition of resistance can occur, leading to the risk of recurrent infections [6,7]. Thus antifungals which impact new targets and have minimal side effects are urgently needed [7].

In fungi, two-component signal transduction (TCST) systems have been implicated in osmotic and oxidative stress responses, cell-cycle control, red/far-red light responses, and virulence switches from non-pathogenic to pathogenic states [8-10]. Since TCST systems are absent in mammalian cells, they are attractive targets for the development of new antifungals with probably minimal side effects in humans [7].

Typical TCST systems in fungi include a histidine kinase (HK), a histidine phosphotransfer protein (HPT) and a response regulator protein (RR). The best understood fungal TCST system is part of the High Osmolarity Glycerol (HOG) pathway in *S. cerevisiae*. In the absence of osmotic stress, the transmembrane HK ScSln1p is active. This HK activity leads to phosphorylation of a histidine residue in the catalytic domain, the so-called HisKA domain, from which the phosphate group is transferred to an aspartic acid residue in an internal receiver domain (REC). Therefore, these HKs are called hybrid HKs. The phosphate group is then shuttled through the HPT protein Ypd1p to the terminal RR proteins Skn7p and Ssk1p [8,11]. Phosphorylated Skn7p is a direct regulator of gene expression, whereas phosphorylated Ssk1p is not able to activate downstream targets. Under conditions of high osmolarity, ScSln1p is inactive, resulting in the dephosphorylation of both RRs. Unphosphorylated Skn7p becomes inactive, whereas unphosphorylated Ssk1p activates a downstream mitogen-activated protein kinase (MAPK) module, in particular the MAP3K Ssk2p resulting in phosphorylation of the MAPK Hog1p [7,12-15]. Phosphorylated Hog1p upregulates the transcription of genes, which encode enzymes that play a key role in glycerol production and maintenance of the intracellular water balance, allowing adaptation to high-osmolarity conditions [13]. Thus, the HK ScSln1p is a negative regulator of the MAPK Hog1p. Likewise, disruption of *ScSLN1* results in the accumulation of unphosphorylated Ssk1p without external stimulus and thus, constitutive activation of Hog1p, which is lethal [14].

**Figure 1:**
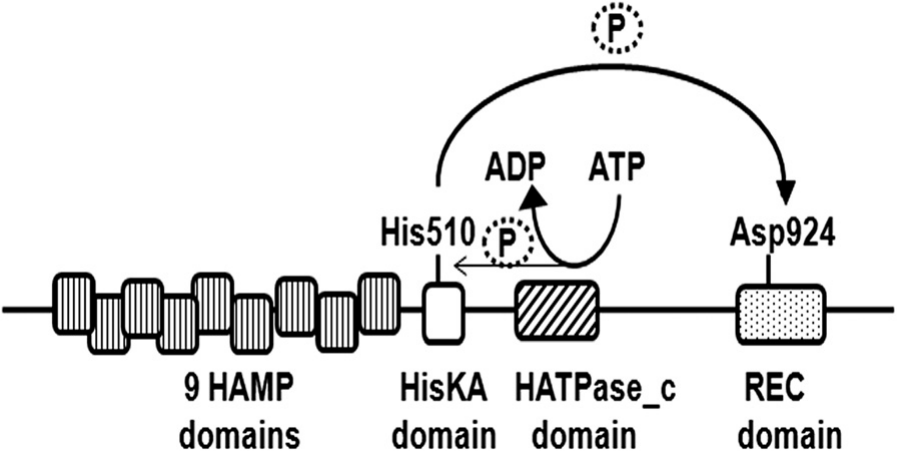
**Schematic representation of the role of different domains of CaNik1p for the protein activity.** ATP binds to the HATPase_c domain, and a phosphate group is first transferred to the conserved phosphate accepting residue His510 of the HisKA domain and then to Asp924 of the REC domain.

While *S. cerevisiae* has a single HK, namely ScSln1p, *C. albicans* has three HKs: CaSln1p, CaNik1p (also called Cos1) and Chk1p [8]. CaNik1p is considered to be a cytosolic enzyme, as it lacks the hydrophobic amino acids indicative of membrane-spanning domains (Figure 1) [16]. The protein is not essential for survival, and a gene deletion mutant could be generated [16-18]. CaNik1p plays an important role in hyphal formation in *C. albicans* on solid media [8,18]. Additionally, the deletion mutant was found to be less virulent in a mouse model for systemic candidiasis [8]. According to the classification scheme of HKs [9], ScSln1p and CaSln1p are group VI HKs while CaNik1p is a group III HK.

Several chemical classes of fungicides, such as phenylpyrroles (fludioxonil), dicarboximides (iprodione) and polyketide secondary metabolites of ambruticins, exert their antifungal effects by activating the HOG signaling pathway, resulting in the accumulation of both glycerol and free fatty acids [19-22]. It is assumed that in the absence of high external osmolarity, artificial induction of excess intracellular glycerol causes leakage of cellular contents and ultimately results in cell death [21,22]. Mutations in group III HKs are frequently associated with fungicide resistance [19], showing the relevance of these enzymes for fungicide activity and placing also these HKs upstream the MAPK Hog1p. It is still discussed, whether group III HKs are negative (as is ScSln1p) [23] or positive [24] regulators of Hog1p. *S. cerevisiae* lacks group III HKs and is usually resistant to the fungicides mentioned above. However, fungicidal sensitivity is gained by heterologous functional expression of group III HKs in *S. cerevisiae* correlating with Hog1p phosphorylation [25-28].

All classes of HKs share the conserved phosphate-accepting domains HisKA, REC and an ATP-binding domain called HATPase_c domain. Based on homology with other HKs, the histidine residue 510 and the aspartate residue 924 in CaNik1p were identified as the essential phosphorylatable residues for the HisKA and the REC domains respectively (Figure 1) [17]. The ATP-binding domain comprises a characteristic N-box with two asparagine residues, which are N623 and N627 in CaNik1p [17]. The N-box is known to be essential for ATP binding [29] and deletion of a single asparagine residue was associated with complete inhibition of ATP binding in the HK EnvZ [30].

Group III HKs are characterized by additional amino acid repeats in the N-terminal part with a length of approximately 90 amino acids each. The repeats contain evolutionary conserved amino acid sequences called HAMP domains. Such abbreviation is due to the frequent occurrence of such domains in histidine kinases, adenylcyclases, methyl accepting chemotaxis proteins and phosphatases, which are proteins associated with signal transduction in both prokaryotic and lower eukaryotic organisms [31]. More than 26400 proteins with HAMP domains exist in the SMART data base. These domains were shown to play an active role in intramolecular signal transduction in prokaryotic sensor kinases. They are composed of about 50 amino acid residues each with two amphipathic helices [32-34] which probably rotate when the sensor domain of the protein is activated as recently elucidated from NMR analysis [35,36]. Unlike the bacterial HK, which usually possess a single HAMP domain, fungal group III HKs have several consecutive HAMP domains. In the five N-terminal amino acid repeats of CaNik1p [16-18] we identified nine HAMP domains of a concatenated structure forming four pairs each with an overall length of 92 amino acids and a single HAMP domain in the remaining truncated amino acid repeat [25].

To study the role of the various protein domains in the function of group III HKs different protein mutants were constructed. In Hik1p, a group III HK from *Magnaporthe grisea,* phosphate acceptance on both the conserved histidine and aspartic acid residues in the catalytic and the receiver domains respectively was essential for the susceptibility to phenylpyrroles and ambruticin VS4 [26,27]. Deletions of single pairs of HAMP domains from the HK CaNik1p of *C. albicans* were associated with decreased susceptibility to fungicides, showing the relevance of these domains for fungicide activity [25] and deletion of four out of five amino acid repeats from the HK DhNik1p of *Dabaryomyces hansenii* generated a constitutively active HK, which was resistant to osmotic stress and fungicide treatment [23,37].

As *C. albicans* is a human pathogen, understanding the relevance of the N-terminal nine HAMP domains and of the HisKA, HATPase_c and REC domains of CaNik1p for the action of antifungal compounds can guide development of new antimycotic strategies. To achieve this goal, point mutations were introduced in the HisKA, HATPase_c and REC domains of *CaNIK1* which should render these domains non-functional. In addition, a deletion mutant for all HAMP domains was generated. Due to the ease of genetic manipulation of *S. cerevisiae* the plasmids harboring the mutated *CaNIK1* were used to transform *S. cerevisiae* followed by testing viability, sensitivity to fungicides and phosphorylation of the MAPK Hog1p upon fungicidal treatment.

## Methods

### Organisms and growth conditions

*S. cerevisiae* BWG1-7a [38] and BY4741 [39] were used in the present study (Table 1).

**Table 1:** ***S. cerevisiae *strains used in this study**

**Strain designation**	**Genotype**	**Transformed with**	**Reference**
BWG1-7a	*Mat* a *ura3-52 leu2-3,112 his4-519 ade1-100*	-	[38]
YES	BWG1-7a	pYES2	This study
NIK	BWG1-7a	pYES2-*CaNIK1-TAG*	[25]
H510	BWG1-7a	pYES2-*CaNIK1(H510Q)*	This study
D924	BWG1-7a	pYES2-*CaNIK1(D924N)*	This study
N627	BWG1-7a	pYES2-*CaNIK1(N627D)*	This study
ΔHa	BWG1-7a	pYES2-*CaNIK1*Δ*HAMP*	This study
ΔHaH510	BWG1-7a	pYES2-*CaNIK1*Δ*HAMP(H510Q)*	This study
ΔH3H4	BWG1-7a	pYES2-*CaNIK1*Δ224-*315*Δ*327-418*aa	[27]
BY4741	*Mat* a *his3*Δ 1; *leu2*Δ 0; *met15*Δ 0; *ura3*Δ 0	-	[39]
ΔHb	BY4741	pYES2-*CaNIK1*Δ*HAMP*	This study
ΔHbH510	BY4741	pYES2-*CaNIK1*Δ*HAMP(H510Q)*	This study
Δssk1	BY4741, *YLR006c::kanMX4*	***-***	[49]
Δpbs2	BY4741, *YJL128c::kanMX4*	***-***	[49]
Δhog	BY4741, *YLR113w::kanMX4*	***-***	[49]
ΔHbΔssk1	Δ*ssk1*	pYES2-*CaNIK1*Δ*HAMP*	This study
ΔHbΔpbs2	Δ*pbs2*	pYES2-*CaNIK1*Δ*HAMP*	This study
ΔHbΔhog	Δ*hog*	pYES2-*CaNIK1*Δ*HAMP*	This study

Prior to transformation, *S. cerevisiae* was grown in YPD medium (Sigma-Aldrich) at 30°C. *S. cerevisiae* transformants were selected and maintained in SD-ura (according to [40]), at 30°C. To obtain high cell density before induction of transgene expression, the transformants were cultivated at 30°C in SD-ura for 36 h. To induce transgene expression from the 36 h SD-ura culture, an overnight culture, a preculture (2–3 h) and ultimately a working culture were prepared in SG-ura. For growth of the reference *S. cerevisiae* strain uracil was added at a concentration of 40 mg/l.

Solidified media were prepared by addition of 1.5% bacto agar (Difco).

*E. coli* XL1-Blue growth, transformation and plasmid DNA preparation were performed using standard methods according to the manufacturer’s instructions.

### Mutagenesis of the cloned *CaNIK1* gene in the pYES2 plasmid and expression of the mutated constructs in *S. Cerevisiae* transformants

The plasmid pYES2-*CaNIK1-*TAG [25] was used as a template for all the generated mutants in the present work. It encodes the wild-type CaNik1p protein fused to a HIS/FLAG tag at the C- terminus. Point mutations were introduced in the HisKA (H510Q), HATPase_c (N627D) and REC (D924N) domains using the quick-change site-directed mutagenesis kit (Stratagene). The nucleotide sequences of the primers used, where the nucleotide changes were introduced to lead to the desired mutations, are given in Table 2. The PCR reaction mixture, the amplification program, the digestion with the restriction enzyme DpnI (Stratagene) and the transformation of the competent cells were carried out according to the manufacturer’s instructions.

**Table 2:** Oligonucleotides used in this study

**Primer**	**Sequence (5´-3´)**	**Used in mutation of:**
F2Gln	CTAGCGAACATGTCGCA**A**GAGATACGTACACC	HisKA domain
R2Gln	GGTGTACGTATCTC**T**TGCGACATGTTCGCTAG	
TAsnF	CTTAACTTGGCTGGT**G**ATGCTATTAAGTTTAC	HATPase_c domain
TAsnR	GTAAACTTAATAGCAT**C**ACCAGCCAAGTTAAG	
AAsnF	GATGTGGTGTTGATG**A**ATGTGCAAATGCCTGTAATG	REC domain
AAsnR	CATTACAGGCATTTGCACAT**T**CATCAACACCACATC	
HMPF1	AGGGAATATT***AAGCTT***ATGAACCCCACTAAAAAACCACG	HAMP domains
HMPR1	GTTCGCGTTTTTGGATTTTTCTAG	
HMPF2	TCCAAAAACGCGAACAGGAATACTGCGGCTAGAGAAGCTG	
HMPR2	GCTCGGTACC***AAGCTT***TCAGTGGTGATGGTGATGATGTCC	

Bold bases are those used to introduce the point mutations. Bold italic bases indicate the HindIII restriction sites. Underlined bases are the overlapping sequences recognized by the in-fusion enzyme.

In CaNik1p (1081 aa), all the HAMP domains (63–485 aa) were deleted using the in-fusion HD cloning kit (Clontech). Briefly, the in-fusion enzyme is able to fuse up to four DNA fragments with a linearized vector upon recognizing 15 bp overlapping sequences at their ends. To allow this fusion, the 15 bp overlaps were introduced to the primers which were used to amplify the target fragments.

The pYES2 vector was linearized using the restriction enzyme HindIII and the pYES-*CaNIK1-*TAG vector was used as a template for amplification of the gene fragments. The sequence of *CaNIK1* upstream of the fragment encoding the HAMP domains (1–186 bp) was amplified using the HMPF1 and HMPR1 primers (Table 2). HMPF1 included homologous 15 bp with the end of the linearized vector downstream of the galactose promoter. The *CaNIK1* fragment located downstream the sequence encoding the HAMP domains and extended by the His-FLAG tag (1454–3243 bp) was amplified using the HMPF2 and HMPR2 primers (Table 2). HMPF2 and HMPR2 shared 15 bp homologous stretches with the 172–186 bp fragment of *CaNIK1* and with the other end of the HindIII-linearized pYES2 vector, respectively. HindIII restriction sites were introduced into the sequences of the HMPF1 and HMPR2 primers. After separation of the PCR amplified fragments by electrophoresis on 1.2% agarose gels, the gel pieces carrying the amplification products were excised and the DNA was purified using a gel extraction kit (Qiagen). The purified fragments were ligated into the digested pYES2 vector using the in-fusion enzyme according to the manufacturer’s instructions.

The existence of the introduced mutations was further confirmed by sequencing the generated constructs (Dept. GNA, HZI, Braunschweig) using primers spanning the target fragments. The mutated constructs were used to transform *S. cerevisiae* using the lithium acetate method [40]. Transformants (Table 1) were selected on SD-ura agar plates.

### Susceptibility assays

In 96 well microtiter plates, working cultures of the transformants were incubated in 180 μl SG-ura supplemented with the appropriate concentrations of the antifungals in triplicates for 24 h. The starting OD at 620 nm was 0.03 and measured using a μQuant scanning microplate spectrophotometer (Biotek). To detect growth inhibitory effects, the OD_620nm_ was again measured after 24 h. The antifungals fludioxonil and iprodione were obtained from Fluka, whereas ambruticin VS3 was produced as described and kindly provided by K. Gerth and R. Jansen (HZI, Braunschweig) [41]*.*

### Detection of Hog1p phosphorylation

Phosphorylation of the MAPK Hog1 was investigated in transformants with *CaNIK1* carrying point mutations as previously described [25]. Briefly, from precultures in SG-ura working cultures of the transformants were prepared in SG-ura containing 10 μg/ml fludioxonil with a starting OD_620nm_ of 0.2. Cells were harvested 15 min after the start of the working culture by centrifugation. Sorbitol (1 M) was used as a positive control, as it is known to stimulate phosphorylation of the MAPK Hog1p via the induction of osmotic stress [42].

To avoid Hog1 phosphorylation caused by cold stress [43], cells were directly shock frozen in liquid nitrogen after centrifugation. Frozen cell pellets were mechanically disrupted by grinding with the mini-dismembrator U (B. Braun Biotech, Melsungen, Germany) in the presence of lysis buffer (10 mM sodium phosphate buffer pH 7, supplemented with 5 mM NaCl, 5 mM KCl, protease and phosphatase inhibitors (cOmplete ULTRA, mini, EDTA free and PhosSTOP (Roche)). Protein concentrations of the supernatants were determined using the BCA assay [44].

A total of 5 μg protein per sample was separated by SDS-PAGE (12.5%) and proteins were blotted onto a PVDF membrane. Phosphorylated Hog1 was detected using the combination of an anti-phospho p38 MAPK (Thr180/182) 3D7 rabbit monoclonal antibody (Cell Signaling Technology) with an HRP-conjugated anti-rabbit IgG (Cell Signaling Technology) as secondary antibody. Incubation of the antibodies was followed by the addition of a peroxidase-specific chemiluminescence substrate (ECL; Advance Western Blotting Detection Kit, GE Healthcare). The bound antibodies were removed by treatment with 1xRe-Blot Plus Solution (Millipore) and subsequently total Hog1p was detected using anti-Hog1 (y-215) sc-9079 rabbit polyclonal IgG (Santa Cruz Biotechnology) and the above mentioned secondary antibody followed by visualization with the ECL substrate.

### Detection of phosphorylation of Hog1p in *S. cerevisiae* transformed with *CaNIK1p*ΔHAMP

Due to the growth inhibitory effect resulting from the expression of CaNik1pΔHAMP in the ΔHa strain, phosphorylation of Hog1p was investigated at an early time point after inducing the expression of CaNik1pΔHAMP. Therefore ΔHa was first cultivated in SD-ura until OD_620nm_ = 1. Cells were harvested by centrifugation and transferred to SG-ura (starting OD_620nm_ = 0.2). After incubation at 30°C for 195 and 210 min, samples were centrifuged and treated as previously mentioned for the detection of Hog1p phosphorylation by Western blotting. Fludioxonil was added as an inducer of Hog1 phosphorylation (positive control) after 180 min at a final concentration of 10 μg/ml.

To confirm the expression of CaNik1pΔHAMP, ΔHa cells were harvested after 180 min incubation in SG-ura, disrupted and the FLAG-tagged protein was detected using a HRP-conjugated monoclonal anti-FLAG antibody (Sigma-Aldrich).

## Results

### The conserved domains of CaNik1p were essential for the susceptibility of *S. cerevisiae* transformants to antifungals

**Figure 2:**
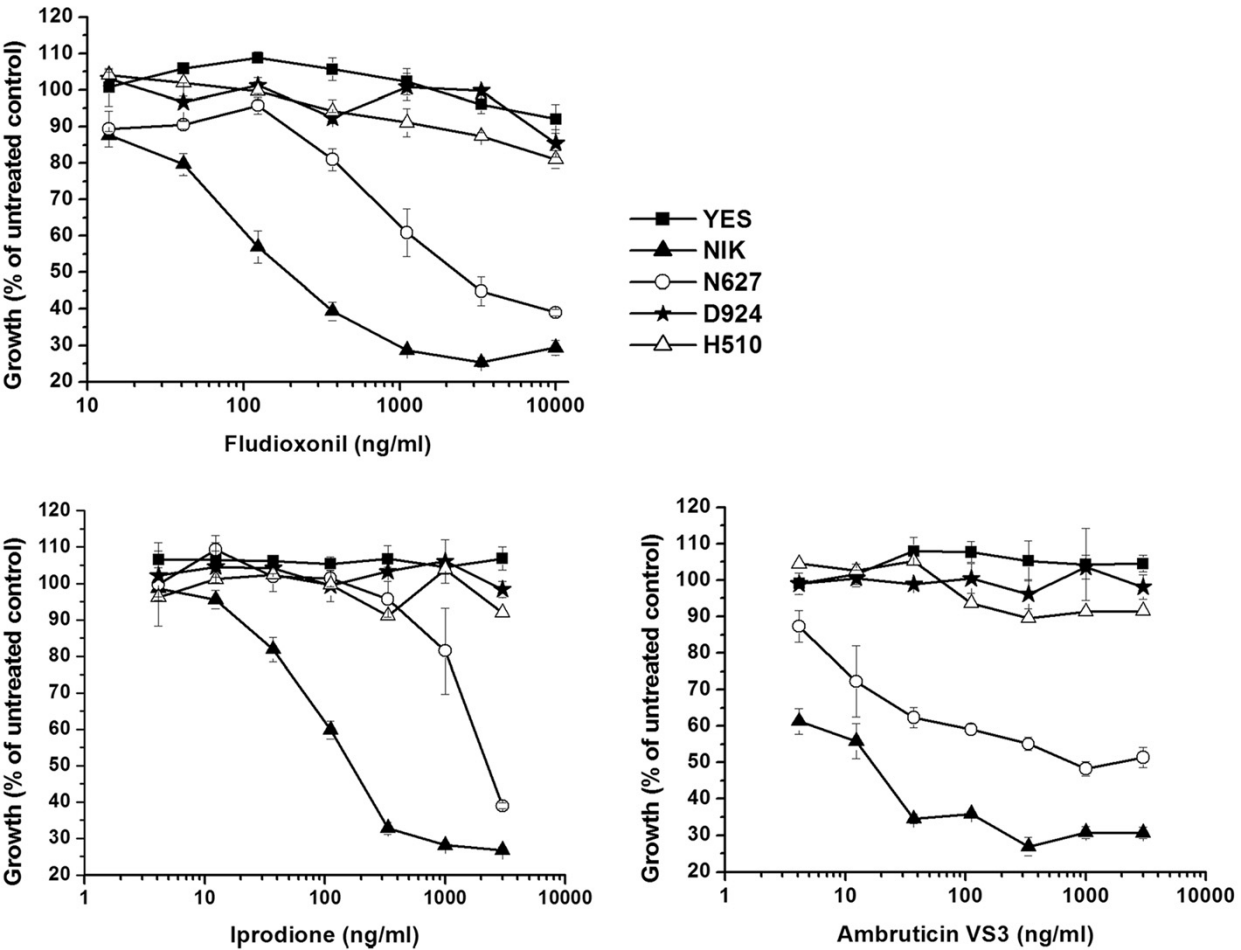
**The conserved domains of CaNik1p were essential for the susceptibility to the fungicides.** The phenylpyrrole fludioxonil, the dicarboximide iprodione and the myxobacterial secondary metabolite ambruticin VS3 were used as representative antifungal compounds targeting fungal group III histidine kinases. Error bars represent the standard deviation from three independent experiments.

After alignment with other HKs, in CaNik1p histidine 510 and aspartate 924 were identified as the essential residues for the HisKA and the REC domains respectively [17] and asparagine 627 for the N-box of the ATP-binding domain. Hence, to inhibit the conserved phosphorylation reactions within CaNik1p, mutant genes were generated, in which either Asn627 from the HATPase_c domain was substituted by aspartate (N627D), His510 by glutamine (H510Q) or Asp924 by asparagine (D924N). *S. cerevisiae* was transformed with the plasmids carrying the mutated *CaNIK1* genes, and the resultant transformants were treated with the antifungals fludioxonil, iprodione and ambruticin VS3. As shown in Figure 2, the strain YES transformed with the empty vector was resistant to all fungicides, while the strain NIK was susceptible to the studied antifungals. The H510Q and D924N point mutations in the HisKA and REC domains respectively, led to complete loss of susceptibility, while the N627D substitution in the HATPase_c domain only decreased the susceptibility to the fungicides in comparison to the strain NIK.

His510 and Asp924 are the conserved phosphate-accepting residues in the HisKA and the REC domains, respectively, which are required for kinase function of hybrid HKs. They are phosphorylated by the histidine kinase activity of the protein (His510) and the subsequent phosphate-transfer to the REC domain within the same protein (Asp924). Loss of fungicide susceptibility of the respective mutants suggested that the functionality of both the HisKA and the REC domain was essential for the antifungal activity.

Probably the N627D mutation did not completely prevent ATP binding to the HATPase_c domain and as a result only a partial effect was obtained.

### Functional HisKA, HATPase_c and REC domains were essential for the phosphorylation of Hog1p after fludioxonil treatment

**Figure 3:**
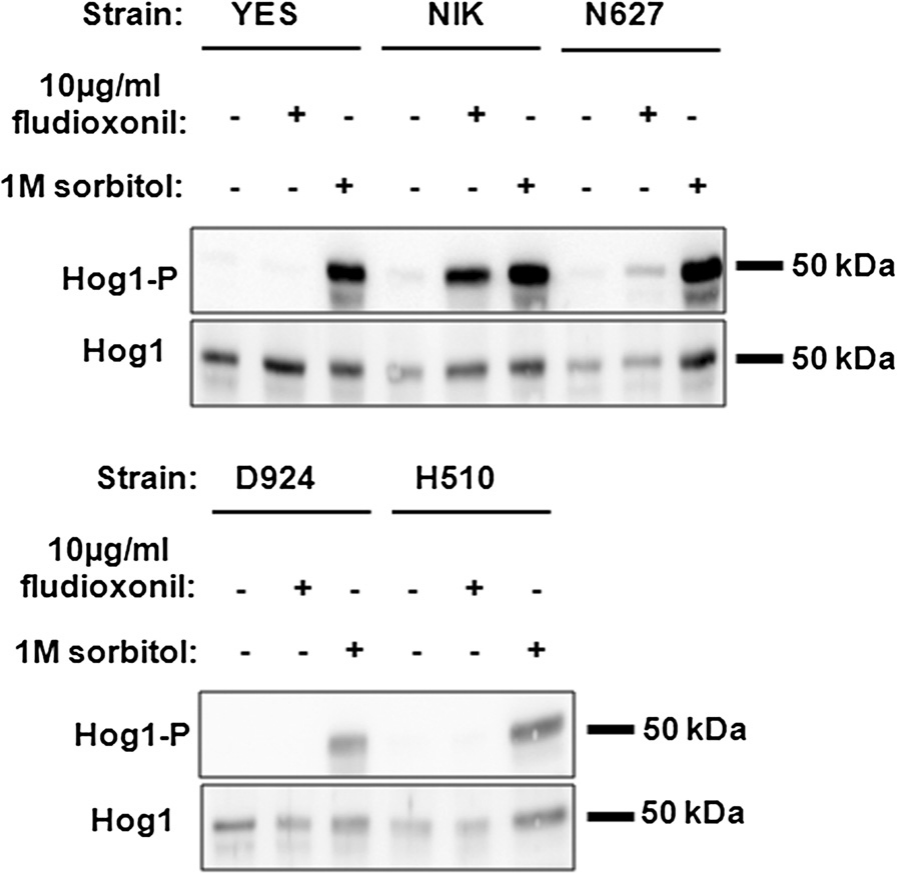
**Hog1p phosphorylation after fludioxonil treatment was dependent on the functionality of conserved domains of CaNik1p.** The phosphorylation of Hog1p (upper panel, Hog1-P) was detected by Western blot after treatment of the strains YES, NIK, N627, D924 and H510 with fludioxonil (10 μg/ml) and sorbitol (1 M), respectively, for 15 min. The presence of Hog1p in all strains was proven (lower panel, Hog1). Hog1p appeared at approximately 50 kDa.

Treatment with fludioxonil led to phosphorylation of the MAPK Hog1p, i.e. to the activation of the HOG pathway, in *S. cerevisiae* transformed with full-length and truncated forms of *CaNIK1*[25]. Therefore, phosphorylation of Hog1p was also analyzed after fludioxonil-treatment of *S. cerevisiae* transformed with *CaNIK1* carrying the H510Q, N627D and D924N point mutations. Phosphorylation of the protein was totally abolished in the mutant strains D924 and H510, while phosphorylation of Hog1p in strain N627 was decreased but not totally inhibited (Figure 3). Thus, activation of Hog1p correlated with the inhibition of the yeast’s growth by fludioxonil and both effects required the functionality of the domains that are essential for the histidine kinase function of the protein, which involves phosphorylation of both His510 and Asp924 of CaNik1p.

Since high concentrations of sorbitol activate the HOG pathway via inhibition of the HK Sln1p, treatment of the transformants with 1 M sorbitol was used as a positive control.

### Normal growth of the yeast was inhibited upon expression of CaNik1pΔHAMP and was restored by inhibition of the HOG pathway

**Figure 4:**
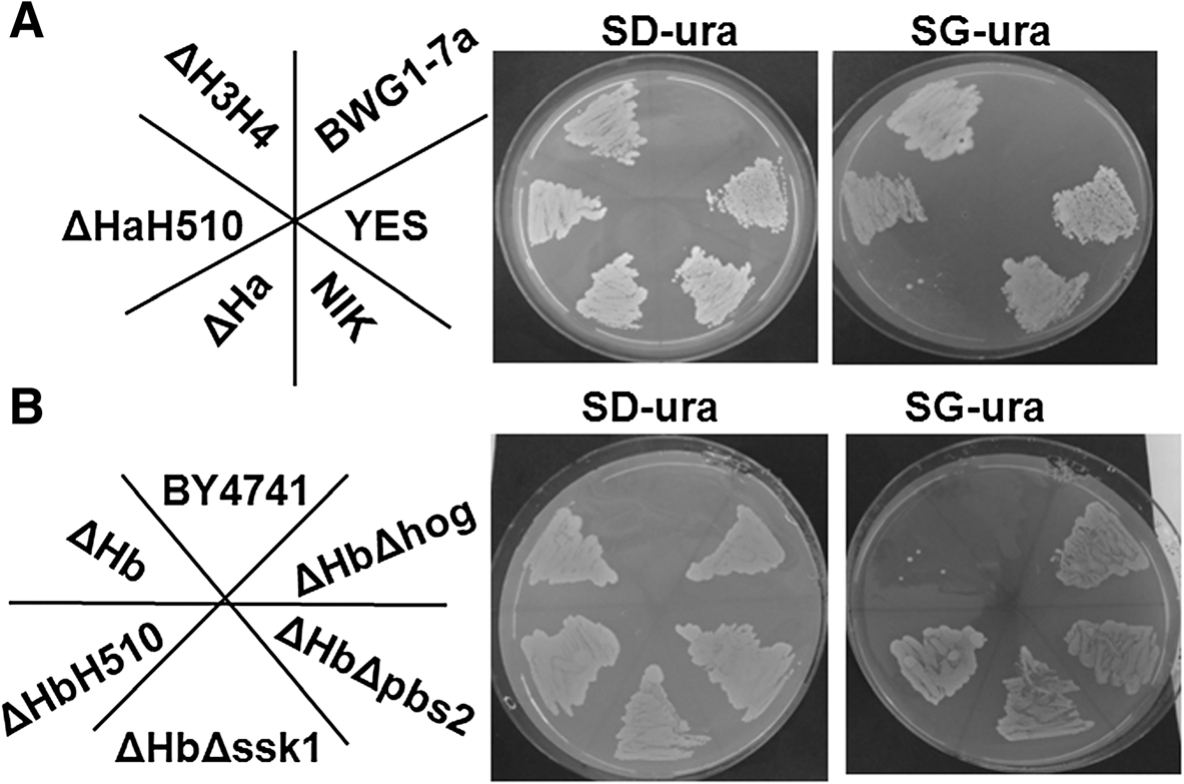
***CaNIK1***Δ***HAMP *expression led to growth inhibition that was dependent on His510 (A) and a functional HOG pathway (B). (A)** Strains BWG1-7a, YES, NIK, ΔHa, ΔHaH510 and ΔH3H4 were streaked on SD-ura and SG-ura agar plates and incubated at 30°C for 4 days. Strain BWG1-7a was the parent strain which is auxotrophic for uracil. **(B)** Strains BY4741, ΔHbΔhog, ΔHbΔpbs2, ΔHbΔssk1, ΔHbH510 and ΔHb were streaked on SD-ura and SG-ura agar plates and incubated at 30°C for 4 days. BY4741 was the parent strain of the single gene deletion mutants, which is auxotrophic for uracil. SG-ura medium induced the expression of the transgenes via the galactose promoter.

Previous work had shown that deletion of single and double pairs of HAMP domains of CaNik1p affected the susceptibility of the resultant mutants to the fungicides [25], and for the HK DhNik1 it was described that deletion of four out of five amino acid repeats generated a constitutively active HK, which could not be inhibited by fludioxonil [23]. Thus we decided to delete all HAMP domains from CaNIK1p. Transforming *S. cerevisiae* with a plasmid carrying a truncated version of *CaNIK1,* in which all HAMP domains were deleted from the protein, resulted in the ΔHa and ΔHb strains (Table 1). These strains were able to grow on SD-ura agar plates, where expression of *CaNIK1ΔHAMP* was not induced. Surprisingly no growth was observed on SG-ura plates, where galactose induced the expression of *CaNIK1ΔHAMP* (Figure 4). This indicated that the presence of *CaNIK1ΔHAMP* had inhibitory effects on the growth of the *S. cerevisiae* transformant, whereas deletion of up to two pairs of HAMP domains did not affect growth of the transformed strain ΔH3H4 [25] (Figure 4A). Simultaneous inactivation of the HisKA domain by the H510Q point mutation restored normal growth of the resultant transformed strains ΔHaH510 and ΔHbH510 (Figure 4).

Limitation of Hog1p activity is essential for the survival of *S. cerevisiae* even under normal growth conditions, as a constitutively active MAP2K Pbs2p, which leads to constitutive activation of Hog1p, is toxic [45]. Thus, we assumed that constitutive activation of Hog1p could be the reason for the growth inhibitory phenotype resulting from the expression of *CaNIK1ΔHAMP*. Therefore, *S. cerevisiae* strains with single gene deletions in the response regulator *SSK1* (strain Δssk1) or components of the Hog1p MAPK module, namely the MAP2K *PBS2* (strain Δpbs2) and the MAPK *HOG*1 (strain Δhog), were transformed with the plasmid pYES2-*CaNIK1ΔHAMP*. These transformants showed normal growth on SG-ura plates (Figure 4B), proving that the growth inhibitory effect associated with the expression of *CaNIK1ΔHAMP* was dependent on the functionality of the HOG pathway.

### Expression of *CaNIK1ΔHAMP* resulted in constitutive phosphorylation of Hog1p that was dependent on the conserved phosphate-accepting histidine residue

**Figure 5:**
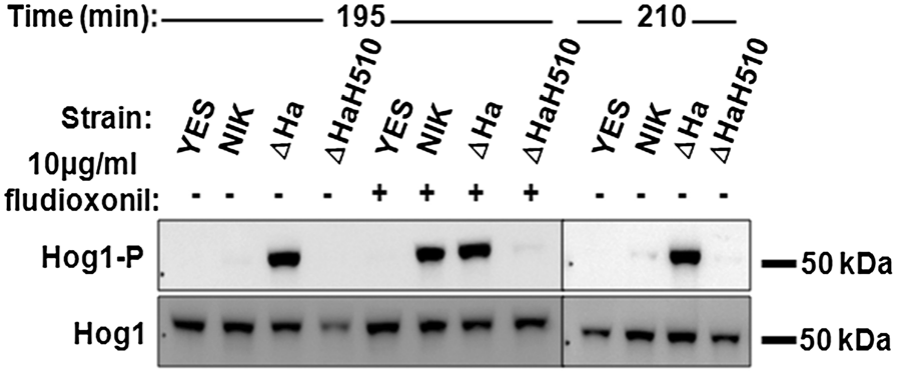
**The MAPK Hog1p was constitutively phosphorylated after expression of *CaNIK1ΔHAMP *in the strain ΔHa.** Phosphorylation of Hog1p (upper panel, Hog1-P) in the strains YES, NIK, ΔHa and ΔHaH510 was detected after cultivation of the strains in SG-ura for 195 and 210 min. Fludioxonil was added after 180 min at a final concentration of 10 μg/ml. The presence of Hog1p (lower panel, Hog1) was confirmed in all strains. Hog1p appears at approximately 50 kDa.

To further analyze the involvement of Hog1p activity, the phosphorylation state of Hog1p was investigated. Due to the growth inhibitory effect resulting from the expression of *CaNIK1ΔHAMP*, the transformant strain ΔHa was first cultivated on the glucose-containing medium SD-ura that does not induce *CaNIK1ΔHAMP* expression to produce sufficient biomass for protein analysis. Subsequently, the expression of *CaNIK1ΔHAMP* was induced by incubating the cells in the galactose-containing medium SG-ura. Gene expression and protein synthesis were allowed for 180 min before fludioxonil was added. Presence of CaNik1pΔHAMP was confirmed by Western blot using an anti-FLAG–antibody (see Additional file 1). Phosphorylation of Hog1p was examined after an additional 15 and 30 min (in total 195 min and 210 min respectively) (Figure 5). After fludioxonil treatment, phosphorylation of Hog1p was observed in the transformant strain NIK1 carrying the full-length protein, and in the transformant strain ΔHa, whereas no phosphorylation was detected in the strains with the empty plasmid (YES) and with the additional H510Q mutation (ΔHaH510), respectively. Hog1p was phosphorylated in the transformant strain ΔHa even without the presence of fludioxonil, while such constitutive phosphorylation was not observed in the strains NIK and ΔHaH510 (Figure 5). Thus, deletion of all HAMP domains from CaNik1p led to constitutive activation of Hog1p without any further external stimulus, which appears to be the reason for the growth inhibitory phenotype of the transformant strain ΔHa in galactose-containing medium.

## Discussion

We previously showed that expression of the group III HK from the human fungal pathogen *C. albicans, CaNIK1* in *S. cerevisiae* resulted in susceptibility of the transformants to the fungicides fludioxonil, iprodione and ambruticin VS3 [25]. Moreover, the fungicidal activity was decreased by deletion of single or double pairs of the N-terminal HAMP domains [25]. For other group III HKs it was already shown that mutations in the conserved phosphate-accepting residues and partial deletion of the HAMP domains conferred fungicide resistance [23,26]. This stimulated our interest to investigate the involvement of the HisKA, HATPase_c and REC domains from CaNik1p in the fungicide activity, as they are conserved in all HKs.

To prevent the primary phosphorylation of the histidine residue and the subsequent His-Asp phosphate-transfer from the HisKA to the REC domains, respectively, the point mutations H510Q and D924N were introduced. The N627D mutation was supposed to inactivate the ATP binding site. The complete resistance of the strains H510 and D924 and the reduced susceptibility of the strain N627 in comparison to the strain NIK clearly showed that the functionalities of the above mentioned domains were essential for the susceptibility of the transformed yeast to the tested fungicides*.* In agreement, similar patterns of Hog1p phosphorylation were obtained after treating the different *S. cerevisiae* transformants with fludioxonil. Phosphorylation of Hog1p was totally abolished in the strains H510 and D924 and partially inhibited in the strain N627, while in all strains expressing genes with point mutations Hog1p was phosphorylated in response to osmotic stress, but was not phosphorylated without external stimuli. These results are in agreement with earlier reports of reduced antifungal susceptibilities of strains, which expressed other group III HKs carrying point mutations in the HisKA and REC domains [26,27]. However, the correlation between the functionality of conserved HisKA, REC and HATPase_c domains of CaNik1p and both the fungicidal sensitivity and phosphorylation of Hog1p after fungicidal treatment was not shown before. Altogether, we present clear evidences that the histidine kinase functionality of CaNik1p was essential for the fungicidal effect and that this effect correlated with the activation of the MAPK Hog1p after treatment with fungicides.

The yeast histidine kinase Sln1p (group VI histidine kinase) is a negative regulator of the MAPK Hog1p, as its inhibition leads to activation of the MAPK. However, for group III HKs different effects were reported: Dic1p, the group III HK from *Cochliobolus heterostrophus,* was described as a positive regulator of Hog1p [24], whereas DhNik1p from *Dabaryomyces hansenii* was identified as a negative regulator [23]. To the best of our knowledge the relationship between the activity of CaNik1p and Hog1p has not yet been investigated, but can now be indirectly deduced from our data. It is generally accepted that activation of Hog1p in the absence of osmotic stress results in growth inhibitory effects [46]. Previously we reported that the antifungal effects of fludioxonil, iprodione and ambruticin VS3 are dependent on the Ssk1 - Pbs2 - Hog1p branch of the osmotic stress response pathway [25], so that a prerequisite for phosphorylation of Hog1p is the non-phosphorylated form of the response regulator Ssk1p [47]. It was even reported that the presence of phosphorylated Ssk1p prevented the activation of the MAP3K Ssk2p from unphosphorylated Ssk1p [48]. Ssk1p receives phosphate groups indirectly from HKs via the histidine transfer protein Ypd1p. Our results indicate that this phosphorylation is inhibited only in strains which are exposed to osmotic stress or which express the wild-type *CaNIK1* variants and are treated with fungicides. In strains expressing mutated non-functional *CaNIK1* phosphorylation of Ssk1 was not inhibited. This conclusion is in agreement with [23] who showed that fludioxonil treatment of *S. cerevisiae* expressing the group III DhNik1p decreased the phosphate transfer to a response regulator even in the presence of the endogenous, active HK Sln1.

**Figure 6:**
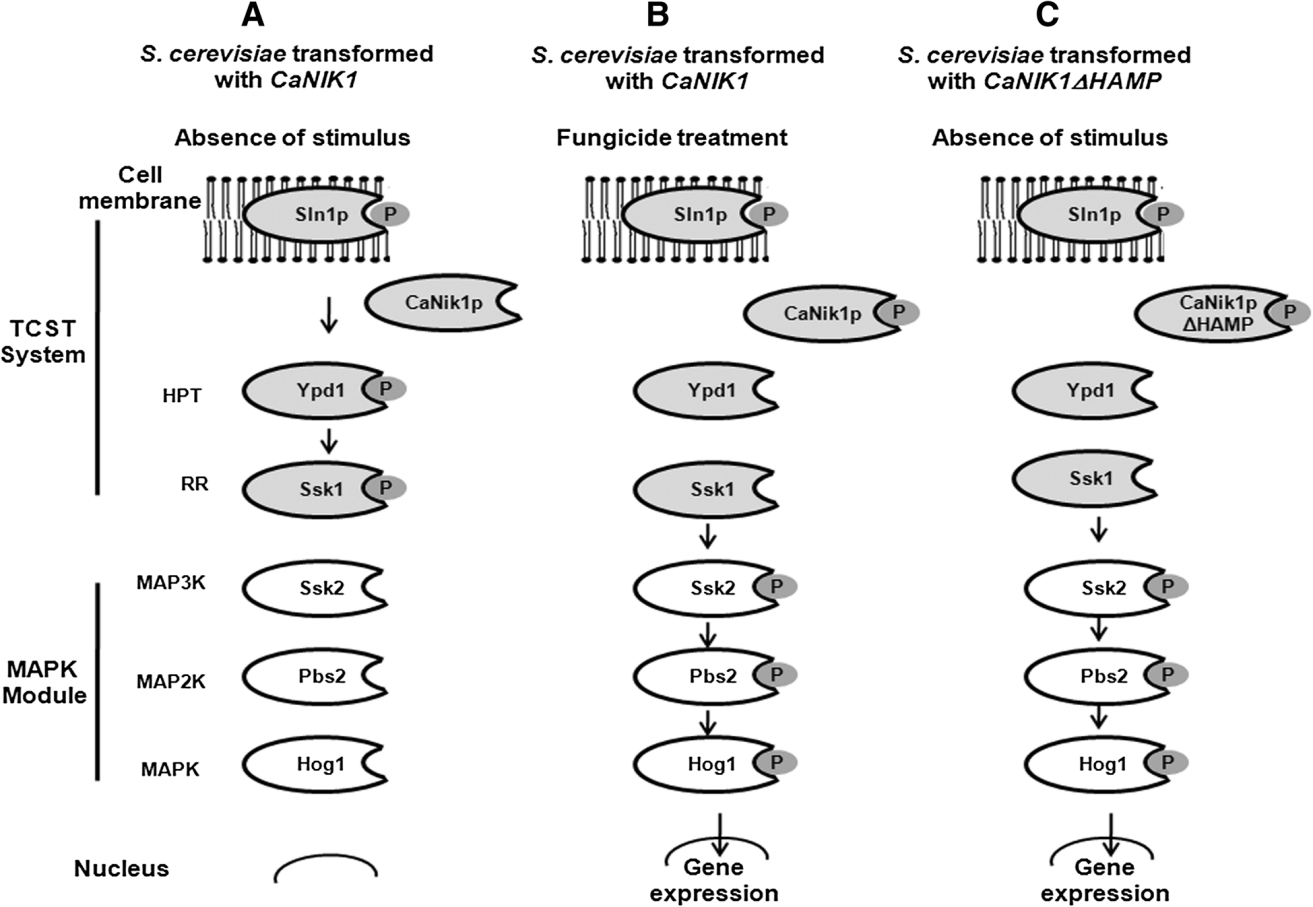
**Model of the activation of the HOG pathway via *CaNIK1 or CaNIK1ΔHAMP, *which were heterologously expressed in *S. cerevisiae.*** In scheme **A**, the initial situation is shown resulting from expression of CaNik1p in *S. cerevisiae*. In scheme **B**, results from fungicide treatment of transformants expressing CaNik1p with point mutations in the conserved domains of histidine kinases were taken into consideration. In scheme **C**, the growth inhibition and the constitutive Hog1 phosphorylation in transformants, in which *CaNIK1ΔHAMP* was expressed, were considered.

Group III HKs are characterized by an amino acid repeat domain with five to six amino acid repeats, in each of which a single HAMP domain was identified previously, but which are now known to comprise concatenated pairs of HAMP domains [25,32,33]. The function of these domains is not yet clear, even though involvement in fungicide susceptibility and in osmosensing were suggested [19,23,25,37]. Previous heterologous expression of truncated proteins, in which several HAMP domains were deleted from group III HKs, i.e. from CaNik1p [25] and DhNik1p from *D. hansenii*[37], was not reported to result in inhibition of growth of the respective *S. cerevisiae* transformants*.* Whereas in the previous reports only selected HAMP domains were deleted, here we deleted all HAMP domains from CaNik1p (CaNik1pΔHAMP) and observed that the synthesis of this truncated protein in the transformed *S. cerevisiae* strain was associated with severe growth inhibition. This phenotype could be reversed by additional point mutation in the histidine phosphorylation site of the HisKA domain (H510) or by the expression of *CaNIK1ΔHAMP* in single gene deletion mutants of the response regulator *SSK1* or of one of the components of the Hog1 module namely the MAP2K *PBS2* and the MAPK *HOG1*. This proved that the inhibition of growth of the transformant upon expression of *CaNIK1ΔHAMP* was dependent on the functionality of both the histidine kinase activity of CaNik1p and the functionality of the Ssk1 - Pbs2 - Hog1 branch of the HOG pathway. We could also unambiguously show that the inhibited growth of the strain ΔHa expressing *CaNIK1ΔHAMP* correlated with the constitutive phosphorylation of Hog1p without any external stimulation, which was also abolished after mutation of the conserved phosphate-accepting histidine residue (*CaNIK1ΔHAMP(H510Q*)). By analogy to the previous discussion this leads to the conclusion that the expression of *CaNIK1ΔHAMP* decreased the phosphate transfer activity to Ssk1, whereas the presence of the mutant CaNik1pΔHAMP(H510Q), which cannot be phosphorylated on the conserved histidine residue, did not affect the endogenous phosphorylation state of the Ssk1p. Thus, in summary, deletion of all HAMP domains had the same effect on the phosphate transfer activity to Ssk1p as treatment with fungicides. Additionally, the presence of mutated proteins, which are assumed not to possess histidine kinase activity and thus are not phosphorylated on either histidine in the HisKA domain or on aspartate in the REC domain, did not inhibit growth of the transformants and did not activate the Hog1p MAPK module. As a consequence of these results, it seems to be unlikely that in the transformed *S. cerevisiae* strains the histidine kinase activity of CaNik1p was inhibited by fungicide treatment, because inhibition of the kinase activity will lead to an enrichment of the non-phosphorylated form of the protein, similar to the protein variants carrying point mutations. The mutated proteins, however, did not influence growth whereas fungicide treatment did. Thus, our results support a model, in which the wild-type CaNik1p protein is not phosphorylated without external stimuli, and Ssk1p is kept in a phosphorylated form via indirect phosphate transfer from Sln1p. Upon deletion of all HAMP domains from CaNik1p or fungicide treatment CaNik1p is phosphorylated and this form prevents phosphate transfer to Ssk1p (Figure 6).

However, this model is based on the assumption that the phosphorylation state of the endogenous histidine kinase Sln1p is not changed by the presence of CaNik1p, since Sln1p is a transmembrane protein that undergoes autophosphorylation in the absence of osmotic stress and CaNik1p is a cystosolic protein. Thus, we expected that CaNik1p does not interfere with the autophosphorylation of the transmembrane protein Sln1p but with the phosphate transfer from Sln1p to Ypd1p. But it may also be hypothesized that when CaNik1p is phosphorylated, either following fungicide exposure or due to the absence of regulatory HAMP domains, Sln1p is no longer phosphorylated leading to the accumulation of non-phosphorylated forms of Ypd1p and Ssk1p and, hence, to the activation of Hog1p.

Thus, CaNik1p has to be considered as a positive regulator of Hog1p activity, similar to Dic1p from *C. heterostrophus* and contrary to DhNik1p and Sln1p*.* This is in agreement with the earlier results that CaNik1p cannot reverse the lethal phenotype of Sln1 deletion in *S. cerevisiae* whereas DhNik1p can. However, the mechanism leading to the reduced overall phosphate transfer activity to the response regulator remains to be investigated. As long as no protein structures are available from group III histidine kinases, one cannot exclude that point mutations and protein truncation have severe effects on the protein structures.

The constructed mutated versions of *CaNIK1* could not be re-integrated in the available *CaNIK1* homozygous deletion mutants of *Candida albicans*[8-18] as these mutants were constructed with the widely used URA blaster method and, thus, are prototrophic for uracil*.* Consequently direct transformation with the pYES2 vectors that harbor the mutated variants of the *CaNIK1* was not possible as the vector contains *URA3* as a selection marker. Therefore a new *CaNIK1* homozygous deletion mutant has to be constructed using for example the *SAT1* flipper cassette that makes use of nourseothricin as an antibiotic selection marker. This will allow reintegration of the *CaNIK1*-mutated variants from this study in such mutant.

## Conclusion

Our results show that functional HisKA, HATPase_c and REC domains of CaNik1p are essential for the antifungal activity of the selected agents activating the HOG pathway. Moreover, the expression of *CaNIK1ΔHAMP* in transformed *S. cerevisiae* was associated with growth inhibition via constitutive phosphorylation of the MAPK Hog1p. In *S. cerevisiae* transformed with *CaNIK1*, growth inhibition resulting from treatment with the selected antifungals or from deletion of all HAMP domains from the protein required both a functional histidine kinase CaNik1p and an intact HOG pathway.

## Abbreviations

HK: Histidine kinase; HOG: High osmolarity glycerol; HPT: Histidine phosphotransfer protein; HRP: Horseradish Peroxidase; MAPK: Mitogen-activated protein kinase; MAP2K: Mitogen-activated protein kinase kinase; MAP3K: Mitogen-activated protein kinase kinase kinase; REC: Receiver domain; RR: Response regulator protein; TCST: Two component signal transduction; SD: Synthetic medium containing dextrose as carbon source; SG: Synthetic medium containing galactose and raffinose as carbon source; Ura: Uracil.

## Competing interests

The authors declare that they have no competing interests.

## Authors’ contributions

MEl-M planned and performed all experiments, presented the results and prepared the manuscript. MMB gave advice for the genetic manipulations, discussed results and contributed to manuscript preparation. UB devised and supervised the whole project, discussed results and prepared the final version of the manuscript. All authors read and approved the final manuscript.

## Supplementary Material

Additional file 1 **Expression of *CaNIK1ΔHAMP *in the strain ΔHa was confirmed after 180 min cultivation in SG-ura.** The strains NIK, ΔHa and ΔHaH510 were cultivated in SG-ura for 180 min before the expression of *CaNIK1*, *CaNIK1*Δ*HAMP* and *CaNIK1*Δ*HAMP (H510Q)*, respectively, was detected in the protein extracts via Western Blot using an anti-Flag antibody. The bands of CaNik1p, CaNik1pΔHAMP and CaNik1pΔHAMP(H510Q) appeared at 121, 75 and 75 kDa respectively. The strain YES with the empty vector was used as control. Click here for file
