# Supplementary figures and images for: Deletion of the HAMP domains from the histidine kinase CaNik1p of Candida albicans or treatment with fungicides activates the MAP kinase Hog1p in S. cerevisiae transformants
Source: BMC Microbiol. 2013 Sep 17;13:209. doi: 10.1186/1471-2180-13-209 (PMC3848655; doi:10.1186/1471-2180-13-209)

kDa

*Marker*

*NIK*

*YES*

$\Delta$ *Ha*

$\Delta$ *HaH510*

150 —

100 —

75 —

50 —

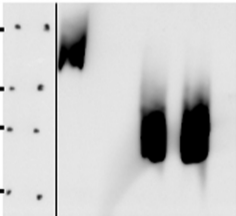

Supplement: Additional file 1 — Expression of CaNIK1ΔHAMP in the strain ΔHa was confirmed after 180 min cultivation in SG-ura. The strains NIK, ΔHa and ΔHaH510 were cultivated in SG-ura for 180 min before the expression of CaNIK1, CaNIK1ΔHAMP and CaNIK1ΔHAMP (H510Q), respectively, was detected in the protein extracts via Western Blot using an anti-Flag antibody. The bands of CaNik1p, CaNik1pΔHAMP and CaNik1pΔHAMP(H510Q) appeared at 121, 75 and 75 kDa respectively. The strain YES with the empty vector was used as control. [file 1471-2180-13-209-S1.pdf]
